# Reinforcement of Dental Methacrylate with Glass Fiber after Heated Silane Application

**DOI:** 10.1155/2014/364398

**Published:** 2014-05-20

**Authors:** Rodrigo Borges Fonseca, Marcella Silva de Paula, Isabella Negro Favarão, Amanda Vessoni Barbosa Kasuya, Letícia Nunes de Almeida, Gustavo Adolfo Martins Mendes, Hugo Lemes Carlo

**Affiliations:** ^1^Department of Operative Dentistry and Dental Materials, Dental School, Federal University of Goiás, Praça Universitária, s/n, Setor Universitário, 74605-220 Goiânia, GO, Brazil; ^2^Department of Restorative Dentistry, Health Sciences Center, Federal University of Paraíba, 58051-900 João Pessoa, PB, Brazil

## Abstract

This study evaluated the influence of silane heat treatment and glass fiber fabrication type, industrially treated (I) or pure (P), on flexural and compressive strength of methacrylate resin bars (BISGMA/TEGDMA, 50/50%). Six groups (*n* = 10) were created: I-sil: I/silanated; P-sil: P-silanated; I-sil/heat: I/silanated heated to 100°; P-sil/heat: P/silanated heated to 100°; (I: I/not silanated; and P: P/not silanated. Specimens were prepared for flexural strength (10 × 2 × 1 mm) and for compressive strength 9.5 × 5.5 × 3 mm) and tested at 0.5 mm/min. Statistical analysis demonstrated the following for flexural strength (*P* < 0.05): I-sil: 155.89 ± 45.27^BC^; P-sil: 155.89 ± 45.27^BC^; I-sil/heat: 130.20 ± 22.11^C^; P-sil/heat: 169.86 ± 50.29^AB^; I: 131.87 ± 15.86^C^. For compressive strength, the following are demonstrated: I-sil: 1367.25 ± 188.77^ab^; P-sil: 867.61 ± 102.76^d^; I-sil/heat: 1162.98 ± 222.07^c^; P-sil/heat: 1499.35 ± 339.06^a^; and I: 1245.78 ± 211.16^bc^. Due to the impossibility of incorporating the stipulated amount of fiber, P group was excluded. Glass fiber treatment with heated silane enhanced flexural and compressive strength of a reinforced dental methacrylate.

## 1. Introduction

Fiber reinforced composites properties can be manipulated according to the required use, creating the possibility of changes in position, orientation, and quantity of inserted fibers. Engineering sciences have developed composites with inclusion of strong fibers within a binder matrix [1]; glass fibers are used by various industries such as aerospace, automotive, and boating [2]. Over the past 30 years, several studies have been undertaken to strengthen dental polymers with various types of fibers, including glass fibers [3–6]. The basic purpose of using fibers in dentistry is to improve the mechanical properties of resins, expanding the possibilities of use and restorative methods [7, 8].

Fiber reinforcement of restorative dental materials [9–12] and endodontic posts [13], its association with inorganic particles [14], its effects on polymerization [15, 16] and mechanical properties of composites [17] have been studied, showing promising results. The effectiveness of glass fiber reinforcement depends on length of fibers [1, 4, 11, 18], form of fibers [17, 19], diameter of the fibers [7, 20], quantity of fibers in the polymer matrix [4, 18], location and direction of the fibers [1, 7, 21], and adhesion of fiber to the polymer matrix [21–23].

In 2006, Garoushi et al. [4] evaluated the effect of length and volume of short fibers (from 1 mm to 5 mm) on the mechanical properties of short glass fibers reinforced composites and observed high values of flexural and compressive strength for samples that employed 22.5 wt% of fibers with 3 mm length. The use of short fibers randomly distributed in the resin matrix promotes multidirectional isotropic reinforcement [13]. Garoushi et al. [4, 9, 13] showed that resin materials reinforced with short glass fiber, randomly distributed, obtained higher values of flexural strength, fracture toughness, and compressive strength.

In order to improve the adhesion between polymer and glass fibers, a silane coupling agent has been used for several decades [24]. Adhesion and impregnation of the fibers to the polymer matrix affect the degree of reinforcement [25], since this promotes effective stress transference from fibers to the polymer matrix [26]. Silanated glass fibers have a higher surface energy and tend to be better impregnated, resulting in better adhesion to polymers [27].

Studies have shown that heat treatment of silanated surfaces at 100°C consolidates the condensation reaction and increases their bonding strength through the elimination of water, alcohol, and other subproducts of the condensation reaction [28]. However, there is no data about the effect of heat treatment of short silanated glass fiber to be used as a reinforcement of composites. It is expected that heat treatment of the silane coupling agent improves the mechanical properties of fiber reinforced composites.

The aim of this study was to evaluate the influence of silane heat treatment and the short glass fiber fabrication type (industrially treated or pure) on compressive and flexural strength of methacrylate resin bars.

## 2. Material and Methods

### 2.1. Experimental Groups

Six experimental groups (*N* = 10) were created according to the interaction of factors in study (2 × 2) plus 2 control groups (Table 1): silane coupling agent application, in two levels (heated or not heated), and short glass fiber fabrication type, in two levels (I: industrially treated; P: pure). For all groups, the reinforced experimental composite (EC) was created with 30% fiber and 70% (in wt%) resin matrix.

The materials used and their respective manufacturers are listed in Table 2. The methacrylate-based resin composition was set as 50 wt% of 2,2-bis ([4-(2-hydroxy-3-metilacriloxipropoxi)phenyl]-propane) (Bis-GMA, Sigma-Aldrich) and 50 wt% of triethylene glycol dimethacrylate (TEGDMA, Sigma-Aldrich) in a photoinitiator system with camphorquinone 1 mol% to 2 mol% of dimethylaminoethyl methacrylate (DMAEMA, Sigma-Aldrich) and 0.1 mol% of butylated hydroxytoluene (BHT, Sigma-Aldrich), which was mixed in a mechanical homogenizer (Model ANS-000, SBS).

The fibers with industrially treated treatment had 3 mm length (Owens Corning, Ribeirão Claro, SP, Brazil). Pure fibers were cut to the same length. In I-sil, P-sil, I-sil/heat, and P-sil/heat groups, fibers received surface treatment with silane coupling agent. After the silanization, the fibers of I-sil/heat and P-sil/heat groups were heat treated at 100°C by an air blow dryer for one minute and then manually incorporated into the resin according to the experimental groups.

### 2.2. Compressive Strength Test

A condensation silicon impression material (Clonage; Nova DFL, Rio de Janeiro, RJ, Brazil) mold was made from a stainless pattern to produce standardized rectangular specimens with dimensions of 9.5 mm × 5.5 mm × 3 mm, according to Garoushi et al. [4]. The EC was inserted into the mold and light polymerized (Radii, SDI, Australia) at 850 mW/cm² for 40 seconds at top and bottom surfaces. The specimens (*n* = 10) were stored in distilled water at 37°C for 24 hours before testing. A compressive load was applied at the center of the specimens to obtain the compressive strength at a crosshead speed of 0.5 mm/min, in a universal testing machine (Instron 5965, Instron Corporation, Canton, MA, USA), and the maximum load to break was recorded in *N*.

### 2.3. Flexural Strength Test

A condensation silicon impression material (Clonage; Nova DFL, Rio de Janeiro, RJ, Brazil) mold was made from a stainless pattern to produce standardized rectangular specimens with dimension of 10 mm × 2 mm × 1 mm, according to Pick et al. [29]. The EC was inserted into the mold and overlaid with polyester strip and then light polymerized (Radii, SDI, Australia) at 850 mW/cm² for 40 seconds at top surface.

The specimens were stored in distilled water at 37°C for 24 hours prior to test, and after that they were positioned on a 3-point bending flexural strength testing apparatus (Instron 5965) with two supports 20 mm apart and tested at a crosshead speed of 0.5 mm/min. The flexure strength (FS) was calculated in MPa with the following equation: FS = 2*PL*/*wb* ^2^, where “*P*” is the maximum load at fracture, “*L*” is the distance between the supports (20 mm), “*w*” is the sample thickness, and “*b*” is the height. The samples' thickness and height were measured with a digital caliper (Mitutoyo, Japan).

### 2.4. Statistical Analysis of Data

A factorial analysis was performed on a 2 × 2 design with a general linear model procedure, excluding control groups and comparing factors in study: silane coupling agent application, in two levels (heated or not heated), and short glass fiber fabrication type, in two levels (industrially treated and pure). After that, data were first submitted to Kolmogorov-Smirnov test to verify the normality of distribution and subsequently analyzed by ANOVA and Tukey-HSD test for comparisons among groups. All tests were performed at a significance level of 5% in SPSS 20.0 software (IBM, New York, USA).

## 3. Results

The factorial analysis showed significant interaction between factors in study, either for flexural strength (*P* = 0.008) or for compressive strength tests (*P* = 0.0001). ANOVA, following Tukey-HSD test, results for flexural and compressive strength are shown in Table 3. For flexural strength test, P-sil/heat group showed the highest strength value (169.86 ± 50.29), although similar to I-sil group (155.89 ± 45.27). I-sil group, on the other hand, was similar to P-sil (127.80 ± 27.57), I-sil/heat (130.20 ± 22.11), and I (131.87 ± 15.86). For the compressive strength test, P-sil/heat group (1499.35 ± 339.06) also showed the highest result, though statistically similar to I-sil (1367.25 ± 188.77). I-sil group, in turn, showed itself similar to I group (1245.78 ± 211.16), which was statistically similar to I-sil/heat (1162.98 ± 222.07). The lowest compressive strength was observed for P-sil group (867.61 ± 102.76). The P group was excluded from this study because it was not possible to incorporate the stipulated amount of fiber.

## 4. Discussion

Among the various types of fibers used for polymers reinforcement in dental treatments, glass fibers stand out because of their good mechanical properties [3–6]. However, the effectiveness of reinforcement depends on many variables, especially the adhesion of glass fibers to the resin matrix [21–23]. This study evaluated the influence of the heat treatment of silane coupling agent and the short glass fiber fabrication type on the flexural and compressive strength of reinforced methacrylate resin bars. It was initially hypothesized that heat treatment of the silane coupling agent could improve mechanical properties of fiber reinforced composites. The results of the present study showed that silane heat treatment is important but depends on the type of short glass fiber used, which partially confirms the driven hypothesis. It was also supposed that fibers with an industrially treated surface treatment would return better results, but this was not confirmed, and also pure fibers treated with heated silane showed the best results, partially confirming this hypothesis.

In order to fibers act as an effective reinforcement for polymers, the stress transfer from the polymer matrix to the fibers is essential and is achieved when fiber length is equal to or greater than the critical fiber length [9, 13], which for a BISGMA based resin ranges from 0.5 to 1.6 mm [29]. In addition, the reinforcing effect is also based on the individual behavior of each fiber to act as a barrier that limits crack propagation [13]. Short fibers randomly distributed provide an isotropic reinforcement in many directions rather than one direction [14]. Based on this knowledge, the experimental composite used in the present study was reinforced with 3 mm short glass fibers in a multidirectional arrangement in the matrix.

The silane coupling agent facilitates the interaction of the polymer matrix to glass fibers. Silanated glass fibers have a higher surface energy and tend to be better impregnated, resulting in better adhesion to polymers [27]. The results of this study showed that all groups containing silanated fibers (I-sil, P-sil, I-sil/heat, and P-sil/heat) could be incorporated easily into the experimental resin during manipulation. On the other hand, it was not possible to incorporate fibers into the experimental resin in P group, a nonsilanated group. It is possible that pure fibers do not undergo any previous chemical surface treatment, preventing the incorporation of high amounts of fibers due to their low surface energy and, consequently, low wetness by the experimental resin.

Theories have been proposed regarding the function of the silane coupling agent, and it was postulated that the adhesion between the silane and the glass fiber is based on two types of links [30]. One of these connections is a siloxane bridge formed by a condensation reaction between the silanol groups and the silica surface. Simultaneously with this condensation reaction, the carbonyl group of the silanol molecule forms hydrogen bonds [27]. Consequently, the condensation of the silane coupling agent becomes important for adhesion between polymer and glass fibers, which can be obtained by heating at temperatures above room temperature [27].

The heat treatment accelerates the silane condensation chemical interaction between the silane monomer and the fiber surfaces [31]. Evaporation of solvents, such as water, alcohol, and acetic acid, causes the elimination of hydrogen bonds on the fiber surfaces, thus increasing sites available for reaction with silane [32]. The silane application is seen as a sensitive technique [33]. Among the factors that affect their effectiveness, evaporation of the solvents plays an important role since incomplete evaporation of these solvents may compromise adhesive action [34].

The results of this study corroborate with this finding, since P-sil/heat with pure fibers being silanated and heated presented the highest values of flexural and compressive strength. However, the same results were not observed for I-sil/heat, in which industrially treated fibers were also silanated and heated. It seems that the previous industrial surface treatment (not disclosed by the manufacturer) negatively reacts with heated silane application. Based on this fact, it is suggested that such treatment on these fibers affects the fiber-to-resin adhesion when subjected to heat treatment, generating low values of flexural and compressive strength.

The major limitation of this study was the difficulty of manipulation of the experimental resin due to the absence of filler particles. The created experimental composite had high fluidity, being difficult to handle. Further studies are needed to improve the experimental properties of this composite. Also the increase of number of specimens per group could have returned a more significant result, reducing standard deviation; in spite of that, all tests showed significant results, which support the following conclusions.

## 5. Conclusions

Within the limitations of this study, it is possible to conclude that the heat treatment of the silane coupling agent is significant to increase the flexural and compressive strength of a dental methacrylate polymer reinforced with pure short glass fibers.

## Figures and Tables

**Table 1:** Experimental groups.

Groups	Description
I-sil	Silanated short glass fibers (industrially treated)
P-sil	Silanated short glass fibers (pure)
I-sil/heat	Silanated and heated (100° for 1 minute) short glass fibers (industrially treated)
P-sil/heat	Silanated and heated (100° for 1 minute) short glass fibers (pure)
I	Nonsilanated short glass fibers (industrially treated)
P	Nonsilanated short glass fibers (pure)

**Table 2:** Materials used in the study.

Material	Manufacturer	Batch
Pure short glass fiber	Maxi Rubber, Diadema, SP, Brazil	∗∗
Industrially treated short glass fiber	Owens Corning, Ribeirão Claro, SP, Brazil	3552
Coupling agent (silane)	Angelus, Londrina, PR, Brazil	24898
Condensation silicon impression material (Clonage)	Nova DFL, Rio de Janeiro, RJ, Brazil	4579
Silicon Carbide Sandpaper 600, 1000, and 1200	Norton Abrasivos, Guarulhos, SP, Brazil	∗∗
BIS-GMA: 2,2-bis ([4-(2-hydroxy-3-metilacriloxipropoxi)phenyl]-propane)	Sigma-Aldrich, St. Louis, MO, USA	MKBK4290V
TEGDMA: triethylene glycol dimethacrylate	Sigma-Aldrich, St. Louis, MO, USA	STBC51937
Camphorquinone	Sigma-Aldrich, St. Louis, MO, USA	STBC7007V
Dimethylaminoethyl methacrylate	Sigma-Aldrich, St. Louis, MO, USA	BCBJ3899V
Butylated hydroxytoluene	Sigma-Aldrich, St. Louis, MO, USA	MKBL3562V

**Table 3:** Mean and standard deviation for flexural strength (MPa) and compressive strength (*N*) for all experimental groups. Statistical comparisons by ANOVA and Tukey (*α* = 0.05) tests.

Groups	Flexural strength	Compressive strength
	(Mean ± SD)	(Mean ± SD)
I-sil	155.89 ± 45.27^BC^	1367.25 ± 188.77^AB^
P-sil	127.80 ± 27.57^C^	867.61 ± 102.76^D^
I-sil/heat	130.20 ± 22.11^C^	1162.98 ± 222.07^C^
P-sil/heat	169.86 ± 50.29^AB^	1499.35 ± 339.06^A^
I	131.87 ± 15.86^C^	1245.78 ± 211.16^BC^

*Different letters mean statistically significant difference for each test, with *P* < 0.05.

**SD: standard deviation.
